# Role of ultrasound in predicting telomerase reverse transcriptase (TERT) promoter mutation in follicular thyroid carcinoma

**DOI:** 10.1038/s41598-024-66351-z

**Published:** 2024-07-03

**Authors:** Myoung Kyoung Kim, Hyunju Park, Young Lyun Oh, Jung Hee Shin, Tae Hyuk Kim, Soo Yeon Hahn

**Affiliations:** 1 Department of Radiology and Center for Imaging Science, Thyroid Center, Samsung Medical Center, Sungkyunkwan University School of Medicine, Seoul, Republic of Korea; 2 Department of Internal Medicine, CHA Bundang Medical Center, CHA University School of Medicine, Seongnam, Republic of Korea; 3 Department of Pathology, Thyroid Center, Samsung Medical Center, Sungkyunkwan University School of Medicine, Seoul, Republic of Korea; 4 Department of Medicine, Thyroid Center, Samsung Medical Center, Sungkyunkwan University School of Medicine, Seoul, Republic of Korea; 5 Department of Radiology and Center for Imaging Science, Thyroid Center, Samsung Medical Center, Sungkyunkwan University School of Medicine, 81 Irwon-Ro, Gangnam-Gu, Seoul, 06351 Republic of Korea

**Keywords:** Follicular thyroid carcinoma, Ultrasound, Telomerase reverse transcriptase promoter mutation, Thyroid Imaging Reporting and Data System, Cancer imaging, Endocrine cancer

## Abstract

Telomerase reverse transcriptase (TERT) promoter mutations are associated with tumor aggressiveness. This study aimed to demonstrate the ultrasonographic (US) features of TERT promoter-mutated follicular thyroid cancer (FTC) and evaluate their predictive performance. A total of 63 patients with surgically confirmed FTC between August 1995 and April 2021 were included. All data were available for analysis of preoperative US findings and TERT promoter mutation results. Genomic DNA was extracted from the archived surgical specimens to identify TERT promoter mutations. Logistic regression analysis was performed to compare US findings between TERT promoter-mutated and wild-type FTCs. Of the 63 patients with FTC, 10 (15.9%) had TERT promoter mutations. TERT promoter-mutated FTCs demonstrated significantly different US suspicion categories compared to wild-type FTCs (*P*s = 0.0054 for K-TIRADS and 0.0208 for ACR-TIRADS), with a trend toward an increasing prevalence of the high suspicion category (40.0% for both K-TIRADS and ACR-TIRADS; *P*s for trend = 0.0030 for K-TIRADS and 0.0032 for ACR-TIRADS). Microlobulated margins and punctate echogenic foci were independent risk factors associated with TERT promoter mutation in FTC (odds ratio = 9.693, 95% confidence interval = 1.666–56.401, *p* = 0.0115 for margins; odds ratio = 8.033, 95% confidence interval = 1.424–45.309, *p* = 0.0182 for punctate echogenic foci). There were no significant differences in the composition and echogenicity of the TERT promoter-mutated and wild-type FTCs. TERT promoter-mutated FTCs were categorized more frequently as high suspicion by the K-TIRADS and ACR-TIRADS. Based on US findings, the independent risk factors for TERT promoter mutations in FTC are microlobulated margins and punctate echogenic foci.

## Introduction

Follicular thyroid carcinoma (FTC) is the second most common cancer after papillary thyroid carcinoma (PTC), accounting for 10% of thyroid malignancies^1^. However, patients with FTCs have higher mortality rates than those with PTCs^2,3^ and appear at a more advanced stage at the time of diagnosis. This is because FTCs exhibit bone, lung, or soft tissue metastases due to their higher vascularity and a higher rate of vascular invasion than PTCs^4^.

According to previous studies on thyroid cancer, high-resolution ultrasound (US) can characterize thyroid nodules and is the modality of choice for preoperative evaluation^5,6^. In addition, US findings are closely related to the clinical behavior of thyroid cancer and the status of genetic mutations, including those in the telomerase reverse transcriptase (TERT) promoter^7^. TERT promoter mutations have been widely recognized for several years as promising novel diagnostic and prognostic genetic markers of differentiated thyroid cancer (DTC). Previous studies have shown that this mutation is associated with tumor aggressiveness and increased recurrence and mortality^8–11^. Therefore, TERT promoter mutation status can provide information for planning appropriate treatment and management of patients with DTC.

Previously, Kim et al. demonstrated that TERT promoter-mutated PTC could be suggested by the US features of nonparallel orientation and microlobulated margins in patients older than 50 years^12^. However, to the best of our knowledge, no studies have investigated the US features of TERT promoter-mutated FTC. Therefore, the purpose of our study was to demonstrate the US features of FTCs with TERT promoter mutations and evaluate their predictive performance.

## Materials and methods

### Patient selection

The institutional review board of our institution approved this study and waived the requirement for informed consent (IRB no. 2022–06-070). In addition, all methods were performed in accordance with the relevant guidelines and regulations.

We collected patient data from August 1995 to April 2021, during which consecutive 87 pathologically confirmed FTC patients underwent thyroid surgery and had postoperative TERT promoter mutation analyses at our institution. The patients were screened using electronic medical and pathological charts. Among these 87 patients, 24 were excluded because their preoperative US evaluations were unavailable. The final cohort included 63 patients with FTC whose preoperative US images and postoperative TERT promoter mutation results were available for the analysis of the index nodule.

We obtained thyroid tumor specimens for genetic analysis and retrospectively collected medical records and death certificates. All tumor factors, including tumor size, extrathyroidal extension, lymph node metastasis, and pathologic stages by the American Joint Committee on Cancer (AJCC)/TNM staging system were evaluated on the pathologic results after surgery. The WHO classifications were determined by an experienced thyroid pathologist (Y.L.O.) after confirming the status of vascular invasion based on the revised version in 2017.

### TERT promoter mutation analysis

TERT promoter mutation analysis was performed on DNA samples from postoperative surgical specimens using a Qiagen DNA FFPE Tissue Kit (Qiagen, Hilden, Germany) according to the manufacturer’s instructions. This was performed using semi-nested polymerase chain reaction amplification and direct Sanger sequencing of hot spots, as previously described (chr5:1,295,228C > T and chr5:1,295,250C > T, commonly termed C228T and C250T)^13,14^.

### US examinations and image evaluation

All preoperative thyroid US examinations were performed with a 5–12 MHz linear array transducer from the Logiq 700 scanner (General Electric Healthcare, Milwaukee, WI), HDI 5000 scanner (Philips Ultrasound, Bothell, WA), or IU22 scanner (Philips Medical Systems, Bothell, WA) by one of eight radiologists (four faculty members and four fellows) with 1–17 years of experience in thyroid imaging.

All US images were retrospectively reviewed by consensus by two radiologists (S.Y.H. and M.K.K.) who were blinded to TERT promoter mutation status. All FTC nodules were assessed for internal composition, echogenicity, margins, calcifications, and orientation based on the Korean Thyroid Imaging Reporting and Data System (K-TIRADS)^15^ and American College of Radiology (ACR)-TIRADS^16^.

Based on K-TIRADS, internal composition was divided into cystic (no obvious solid component), predominantly cystic (> 50% of the cystic portion), predominantly solid (≤ 50% of the cystic portion), or solid (no cystic component). Echogenicity was classified as hyper-, iso-, mild hypoechogenicity (compared to the echogenicity of the normal thyroid parenchyma), or marked hypoechogenicity (compared to the echogenicity of the adjacent strap muscle). Margins were categorized as smooth, ill-defined, or irregular including spiculated and microlobulated, or extrathyroidal extensions. Calcifications were described as punctate echogenic foci, macrocalcifications, rim or peripheral calcifications, or intracystic echogenic foci with comet-tail artifacts. Orientation was divided into parallel or nonparallel. The final K-TIRADS^15^ assessments were classified into five categories as follows: category 1, no nodule; category 2, benign (iso-/hyperechoic spongiform, partially cystic nodule with intracystic echogenic foci and comet tail artifact, or pure cyst); category 3, low suspicion (partially cystic or iso-/hyperechoic nodule without any of the three suspicious US features: punctate echogenic foci, nonparallel orientation, or irregular margin); category 4, intermediate suspicion (solid hypoechoic nodule without any of the three suspicious US features, partially cystic or iso-/hyperechoic nodule with any of the three suspicious US features, or entirely calcified nodules); category 5, high suspicion (solid hypoechoic nodule with any of the three suspicious US features).

Based on the ACR-TIRADS^16^, points were given for all US features in a nodule and additional points for more suspicious features, as follows: composition, 0 points for cystic or almost completely cystic and spongiform, 1 point for mixed cystic and solid, 2 points for solid or almost completely solid; echogenicity, 0 points for anechoic, 1 point for hyperechoic or isoechoic, 2 points for hypoechoic, 3 points for very hypoechoic; shape, 0 points for wider-the-tall, 3 points for taller-than-wide; margin, 0 points for smooth, 0 points for ill-defined, 2 points for lobulated or irregular, 3 points for extra-thyroidal extension; echogenic foci, 0 points for none or large, 1 point for macrocalcifications, 2 points for peripheral (rim) calcifications, 3 points for punctate echogenic foci. The point total determined the nodule’s ACR-TIRADS level, and the US features were categorized as benign (0 point), not suspicious (2 points), mildly suspicious (3 points), moderately suspicious (4–6 points), or highly suspicious (7 points or more) for malignancy.

### Statistical analysis

We used the χ^2^ or Fisher’s exact test to compare categorical variables such as clinicopathological and US features and the Mann–Whitney U test to compare continuous variables such as age and size in patient groups with and without TERT promoter mutations. Multivariate logistic regression analysis was performed to determine whether US findings were independent variables for predicting TERT promoter mutations. Odds ratios (ORs) and 95% confidence intervals (95% CIs) were calculated. The diagnostic performance of the US findings was assessed by estimating their sensitivity, specificity, and positive and negative predictive values. All statistical data analyses were performed using SPSS software (PASW Statistics, version 27; SPSS, Chicago, Ill, USA), and a *p* value < 0.05 was considered statistically significant.

## Results

### Clinicopathological characteristics

TERT promoter mutations were detected in 15.9% (10/63) of the FTC lesions. There were 46 women (mean age 43.6 ± SD years; age range, 14–75 years) and 17 men (mean age, 43.4 ± SD years; age range, 23–75 years). Mean age of the patients was 43.5 ± SD years (age range, 14–75 years). The mean size of the tumors was 4.15 ± SD cm (size range, 1.1–10.0 cm). Table 1 demonstrates the clinicopathological characteristics of patients with FTC according to TERT promoter mutation status. FTCs with TERT promoter mutations were significantly associated with older age, widely invasive WHO grade, higher AJCC/TNM stage, distant metastasis, recurrence, and death (all *ps* < 0.035). Gross extrathyroidal extension and TNM stage III/IV were found only in the TERT promoter-mutated FTC group. There were no significant differences in sex or primary tumor size between TERT promoter-mutated and wild-type FTC groups.

**Table 1:** Clinicopathological characteristics of follicular thyroid carcinoma according to the tert promoter mutation status.

	TERT promoter wild type FTC (n = 53)	TERT promoter mutated FTC (n = 10)	p-value
Sex (*n*, %)			0.72
Female	38 (71.7)	8 (80.0)	
Male	15 (28.3)	2 (20.0)	
Age, year (mean ± SD)	41.70 ± 13.26	53.90 ± 16.44	0.02*
Age group (*n*, %)			0.02*
< 55 years	45 (54.9)	5 (50.0)	
≥ 55 years	8 (15.1)	5 (50.0)	
Size, cm (mean ± SD)	3.74 ± 1.51	4.86 ± 3.15	0.46
Size group (*n*, %)			0.30
≤ 4 cm	32 (60.4)	4 (40.0)	
> 4 cm	21 (39.6)	6 (60.0)	
WHO classification (*n*, %)			0.02*
MI-FTC	34 (64.2)	3 (30.0)	
EA-FTC	14 (26.4)	3 (30.0)	
WI-FTC	5 (9.4)	4 (40.0)	
Gross ETE (*n*, %)	0	1 (10.0)	0.16
Distant metastasis (*n*, %)	2 (3.8)	3 (30.0)	0.03*
AJCC/TNM 8th stage (*n*, %)			0.01*
Stage I	47 (88.7)	5 (50.0)	
Stage II	6 (11.3)	4 (40.0)	
Stage III/IV	0	1 (10.0)	
Recurrence (*n*, %)	4 (7.5)	5 (50.0)	< 0.01*
Death (*n*, %)	2 (3.8)	3 (30.0)	0.03*

*A *p-*value of < 0.05 was considered statistically significant.

MI-FTC, minimally invasive follicular thyroid carcinoma; EA-FTC, encapsulated angioinvasive follicular thyroid carcinoma; WI-FTC, widely invasive follicular thyroid carcinoma; SD, standard deviation; ETE, extrathyroidal extension; AJCC, American Joint Committee on Cancer; TNM, tumor node metastasis.

### US Imaging findings

**Figure 1:**
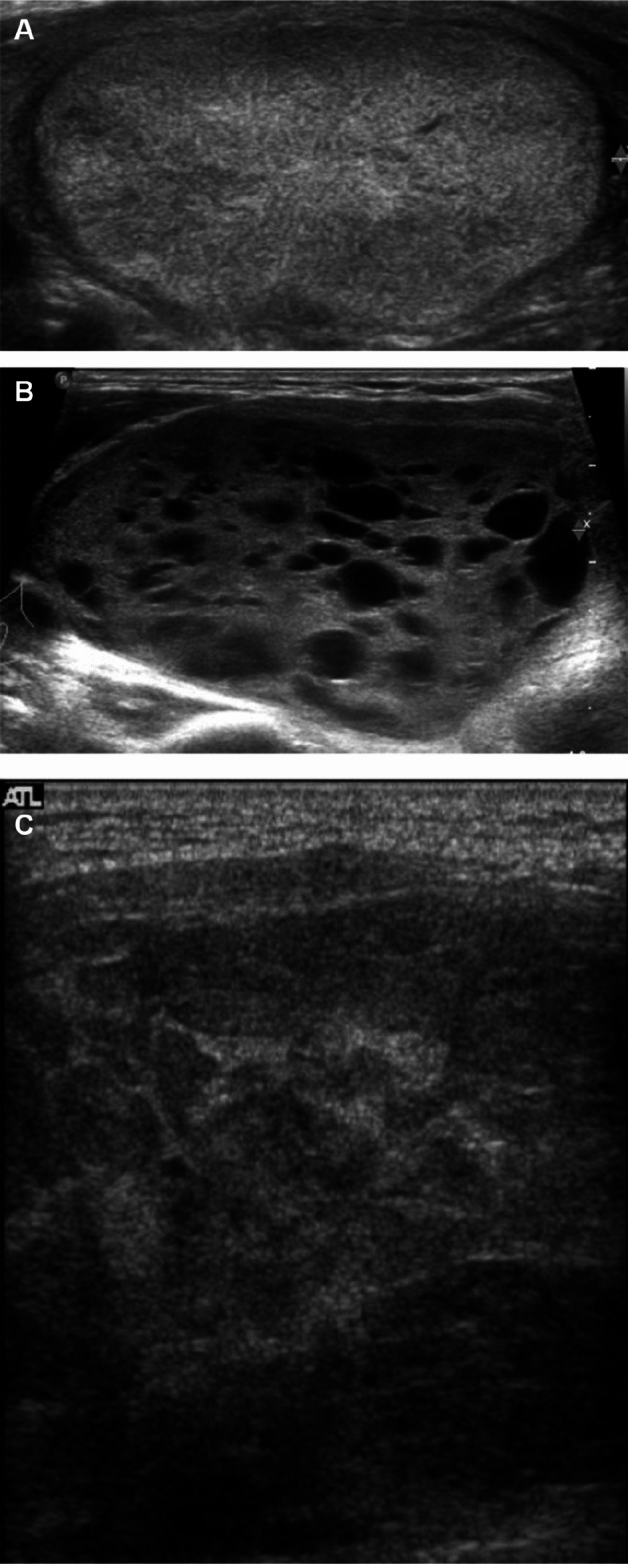
The TERT promoter wild type follicular thyroid carcinomas. Transverse ultrasound images showing a solid isoechoic nodule (**A**), a predominantly solid hypoechoic nodule (**B**), and a solid hypoechoic nodule (**C**) with smooth margins and no calcifications. These were classified as K-TIRADS category 3/ACR-TIRADS category 2 (A), K-TIRADS/ACR-TIRADS category 3 (**B**), and K-TIRADS/ACR-TIRADS category 4 (**C**).

Regardless of the presence of TERT promoter mutations, the most common US findings of FTC were solid nodules (69.8%) with mild hypoechogenicity (50.8%), smooth margins (85.7%), parallel orientation (100%), and no calcification (63.5%) (Fig. 1).

**Figure 2:**
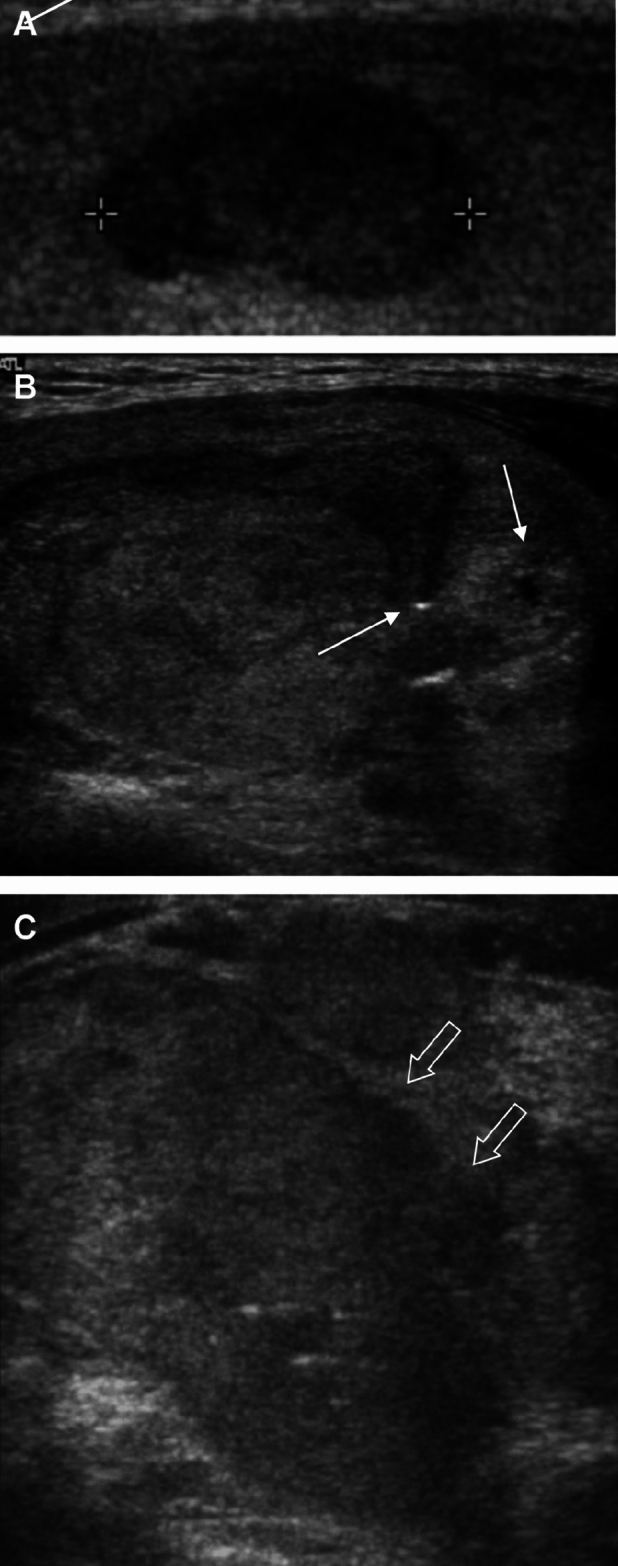
The TERT promoter mutated follicular thyroid carcinomas. Transverse ultrasound images show solid hypoechoic nodules with markedly decreased echogenicity (**A**), punctate echogenic foci (solid arrow) (**B**), or irregular margins (open arrow) (**C**). They were categorized as K-TIRADS/ACR-TIRADS category 4 (**A** and **B**), and K-TIRADS/ACR-TIRADS category 5 (**C**).

In univariate analyses of US features (Table 2), TERT promoter mutations were significantly associated with irregular margins (*p* = 0.0288). In particular, all cases with irregular margins had microlobulated margins. Although the *p*-value approached significance for calcification (*p* = 0.0677), the frequency of punctate echogenic foci in TERT promoter-mutated FTCs was higher than those in wild-type FTCs (40.0 vs. 11.3%; *p* = 0.0439). However, the TERT promoter mutations were not associated with composition, echogenicity, or orientation. TERT promoter-mutated FTCs demonstrated significantly different US suspicion categories compared to wild-type FTCs (*p*s = 0.0054 for K-TIRADS and 0.0208 for ACR-TIRADS), with a trend toward an increasing prevalence of the high suspicion category (40.0% for both K-TIRADS and ACR-TIRADS; *p*s for trend = 0.0030 for K-TIRADS and 0.0032 for ACR-TIRADS). In multivariate analyses (Table 3), irregular margins and punctate echogenic foci were independent risk factors associated with TERT promoter mutations in FTC (odds ratio = 9.693, 95% confidence interval = 1.666–56.401, *p* = 0.0115 for margins; odds ratio = 8.033, 95% confidence interval = 1.424–45.309, *p* = 0.0182 for punctate echogenic foci) (Fig. 2).

**Table 2:** Ultrasound characteristics of follicular thyroid carcinoma according to TERT promoter mutation status.

	TERT promoter wild type FTC (n = 53)	TERT promoter mutated FTC (n = 10)	p-value	p for trend
Composition			0.36	
Predominantly cystic	3 (5.7)	0		
Predominantly solid	15 (28.3)	1 (10.0)		
Solid	35 (66.0)	9 (90.0)		
Echogenicity			0.46	
Iso/Hyperechogenicity	22 (41.5)	3 (30.0)		
Mild hypoechogenicity	27 (50.9)	5 (50.0)		
Marked hypoechogenicity	4 (7.5)	2 (20.0)		
Margins			0.03^a^	
Smooth	48 (90.6)	6 (60.0)		
Irregular (microlobulated)	5 (9.4)	4 (40.0)		
Orientation			1.00	
Parallel	53 (100.0)	10 (100.0)		
Calcification			0.07	
No	36 (67.9)	4 (40.0)		
Punctate echogenic foci	6 (11.3)	4 (40.0)		
Other	11 (20.8)	2 (20.0)		
K-TIRADS			0.01^a^	< 0.01^b^
3 (Low suspicion)	22 (41.5)	0		
4 (Intermediate suspicion)	25 (47.2)	6 (60.0)		
5 (High suspicion)	6 (11.3)	4 (40.0)		
ACR-TIRADS			0.02^a^	< 0.01^b^
2 (Not suspicious)	10 (18.9)	0		
3 (Mildly suspicious)	9 (17.0)	0		
4 (Moderately suspicious)	31 (58.5)	6 (60.0)		
5 (Highly suspicious)	3 (5.7)	4 (40.0)		

^a^A *p* value of < 0.05 was considered statistically significant.

^b^A *p* for trend value of < 0.05 was considered statistically significant.

*ACR* American College of Radiology, *K* Korean, *TIRADS* Thyroid Imaging Reporting and Data System.

**Table 3:** Multivariate analysis of independent ultrasound characteristics for predicting TERT promoter mutations in patients with follicular thyroid carcinoma.

US findings	β Coefficient	Odds ratio	p-value
Irregular (microlobulated) margins	2.271 ± 0.899	9.7 (1.7–56.4)	0.01
Punctate echogenic foci	2.084 ± 0.883	8.0 (1.4–45.3)	0.02

Data in parentheses are 95% confidence intervals.

Estimates of the diagnostic performance of the two US findings in predicting TERT promoter mutations are shown in Table 4. The negative predictive values were 88.9% for the irregular margins and 88.7% for punctate echogenic foci. In addition, the positive predictive values were 44.4% for irregular margins and 40.0% for punctate echogenic foci, and the diagnostic accuracies were 82.5% for irregular margins and 81.0% for punctate echogenic foci.

**Table 4:** Diagnostic performances of ultrasound characteristics in predicting TERT promoter mutations in patients with follicular thyroid carcinoma.

US findings	Sensitivity (%)	Specificity (%)	Positive predictive value (%)	Negative predictive value (%)	Accuracy (%)
Irregular margin	40.0 (12.2–73.8)	90.6 (79.3–96.9)	44.4 (20.6–71.2)	88.9 (82.7–93.0)	82.5 (70.9–91.0)
Punctate echogenic foci	40.0 (12.2–73.8)	88.7 (77.0–95.7)	40.0 (18.6–66.0)	88.7 (82.4–92.9)	81.0 (69.1–89.8)

Data in parentheses are 95% confidence interval.

## Discussion

To the best of our knowledge, no previous study has demonstrated a correlation between US findings and TERT promoter mutations in FTC. Our study showed that US findings suggestive of TERT promoter mutations were microlobulated margins and punctate echogenic foci. Moreover, patients older than 55 years were also associated with TERT promoter-mutated FTC.

Several retrospective studies have described the imaging features of follicular carcinomas using US. Our study showed the results are consistent with those of previous studies. FTCs with microlobulated margins showed a ninefold increase in the risk of TERT promoter mutations according to multivariate analysis of US findings. Pompili et al. reported that an irregular margin of the nodule is significantly correlated with malignancy^17^. Another study showed that the microlobulated margin could be used to predict TERT promoter-mutated PTCs^12^. The irregular margin is presumed as attraction of surrounding tissue by peripheral stromal traction, which may be relevant to the association between TERT promoter mutation and tumor aggressiveness.

Another unique US finding in TERT promoter-mutated FTC is punctate echogenic foci, showed an eightfold increase in the risk of having a TERT promoter mutation. There have been studies in which calcifications were associated with an increased risk of thyroid malignancy^18–20^, which was also observed in our study. Shin et al. compared US findings between widely and minimally invasive follicular thyroid carcinomas, and their results indicated a higher frequency of calcifications in cases of widely invasive follicular carcinoma^21^ The microcalcifications observed in our study showed small in quantity compared with the classic appearance of microcalcifications in PTC. It can be hypothesized that tissue necrosis, hemorrhage, or both, reflect tumor aggressiveness in TERT-promoted FTCs, whereas microcalcifications in PTCs are thought to originate from psammoma bodies^22^.

Hypoechogenicity and the absence of internal cystic changes are associated with an increased risk of follicular neoplasms becoming carcinomas. The rapid proliferation of malignant cells is presumed to cause the loss of normal, orderly follicles in the normal thyroid parenchyma. This is consistent with our results, which showed that the frequency of solid lesions was 66% in wild-type FTCs and 90% in TERT-promoted FTCs. The frequency of hypoechogenicity/marked hypoechogenicity was 58.4% in wild-type FTCs and 70% in TERT-promoted FTCs. However, there was no significant difference in TERT promoter mutation status.

Our study showed that the rate of WI-FTC groups for each wild-type and TERT promoter-mutated groups was 9.4 and 40.0%, respectively. We can assume that the aggressiveness of TERT promoter mutations reflects the presence of vascular invasion according to the WHO classification. This was also reported in a recent study^23^, which showed that increasing tumor invasiveness and worsening prognosis in FTC, based on the WHO classification and TERT promoter mutation results, were positively correlated with US features that indicated malignancy probability according to both K-TIRADS and ACR-TIRADS.

This study had several limitations. First, this was a retrospective study conducted in a single tertiary institution, and we analyzed patients who underwent surgery and revealed TERT promoter mutations in surgical specimens. At our institution, TERT promoter mutation analysis has been routinely performed on all thyroid surgical specimens since 2019, but prior to that, analysis was performed on a limited number of specimens depending on clinical necessity. Therefore, the number of study population may seem relatively small compared to the 25-year study period. Therefore, there may have been a potential selection bias. Second, because of the rarity of FTC in the iodine-sufficient areas of South Korea, our study sample size was small. There may be a potential selection bias due to the above two limitations. Third, advances in US equipment over the more than 25 years of our study may have influenced the resolution and analysis of US images. Further prospective studies with larger sample sizes are warranted to validate our results.

In conclusion, FTCs with TERT promoter mutations were categorized more frequently as high suspicion by K-TIRADS and ACR-TIRADS. Based on US findings, microlobulated margins and punctate echogenic foci were independent risk factors associated with TERT promoter mutations in FTC.

## Data Availability

The datasets used and/or analysed during the current study available from the corresponding author on reasonable request.
